# Obesogenic neighborhood environment is associated with body fat and low-grade inflammation in Brazilian children: could the mother’s BMI be a mediating factor?

**DOI:** 10.1017/S1368980023002628

**Published:** 2023-11-30

**Authors:** Mariana De Santis Filgueiras, Milene Cristine Pessoa, Josefina Bressan, Ariene Silva do Carmo, Aline Siqueira Fogal Vegi, Fernanda Martins de Albuquerque, Juliana Farias de Novaes

**Affiliations:** 1 Department of Nutrition and Health, Universidade Federal de Viçosa, Av. P.H. Rolfs s/n, Centro de Ciências Biológicas II, Campus Universitário, Viçosa, Minas Gerais 36570-900, Brazil; 2 Department of Nutrition, Nursing School, Universidade Federal de Minas Gerais, Av. Prof. Alfredo Balena 190, Santa Efigênia, Belo Horizonte, Minas Gerais 30130-100, Brazil; 3 Nutrition School, Universidade Federal de Ouro Preto, Rua Dois, Campus Morro do Cruzeiro, Ouro Preto, Minas Gerais 35400-000, Brazil; 4 Nutrition Institute, Universidade do Estado do Rio de Janeiro, Rua São Francisco Xavier 524, 12th floor, Maracanã, Rio de Janeiro 20550-900, Brazil

**Keywords:** Adiposity, Adipokines, Inflammation, Built environment, Food environment

## Abstract

**Objective:**

To evaluate the direct and indirect associations of obesogenic and leptogenic neighborhood environments with body fat, and pro- and anti-inflammatory adipokines in Brazilian children.

**Design:**

Cross-sectional study. The body fat distribution was assessed using dual-energy X-ray absorptiometry (DXA). Concentrations of leptin and adiponectin were measured. Four hundred meters (0·25 miles) road network buffer was the neighborhood unit used to assess the environmental characteristics around households. Obesogenic and leptogenic environments were the latent variables obtained from the observed characteristics. The mother’s BMI, ultra-processed food consumption, and physical activity before and after school, were tested as mediating variables. A hybrid model of structural equations was used to test the direct and indirect associations of obesogenic and leptogenic environments with body fat, leptin and adiponectin concentrations.

**Setting:**

Urban area of Viçosa, Minas Gerais, Brazil.

**Participants:**

Children aged 8- and 9-years (*n* 367).

**Results:**

Obesogenic environment was directly associated with the mother’s BMI (*β*: 0·24, *P* = 0·02) and the child’s body fat (*β*: 0·19, *P* = 0·02). The mother’s BMI and body fat mediated the association of the obesogenic environment with leptin concentrations (*β*: 0·05, *P* = 0·02).

**Conclusions:**

Obesogenic neighborhood environment was directly associated with body fat and mother's BMI, and indirectly associated with leptin concentrations in Brazilian children, mediated by the mother’s BMI and body fat.

Overweight and pediatric obesity are considered public health problems, affecting over 340 million children and adolescents worldwide^(1)^. Adipose tissue secretes several peptides, such as adipokines, which are involved in inflammatory and immune functions, cardiovascular homeostasis, among other biological and physiological functions^(2,3)^. Some studies have shown that children with overweight or obesity have higher concentrations of pro-inflammatory adipokines, such as leptin, and lower concentrations of anti-inflammatory adipokines, such as adiponectin^(4–6)^.

The interaction of individual and environmental factors results in childhood obesity and adiposity-related inflammation^(7,8)^. The child’s dietary pattern and practice of physical activity (PA) are considered proximal determinants of body fat^(8)^. Jointly, the unhealthy lifestyle and the higher BMI of parents can influence a child’s behavior (for instance, unhealthy diet and physical inactivity) and excess of weight^(8)^. However, several environmental characteristics may be directly or indirectly related to adiposity markers. An obesogenic environment, whose conditions favor choices for a lifestyle that promotes obesity, such as unhealthy eating and physical inactivity, may influence childhood obesity^(8,9)^. On the other hand, the presence of a leptogenic environment, that is, one that promotes choices for a healthy lifestyle, such as PA and healthy eating, can prevent and/or reduce body fat in children^(8,9)^.

In all age groups, the relationship of obesogenic and leptogenic environments with low-grade inflammatory markers is poorly investigated. In Brazilian children, the higher density of public PA facilities around schools was associated with lower leptin concentrations^(10)^. In addition, obesogenic environmental factors, such as air pollution and exposure to ultra-processed foods (UPF), can promote endocrine dysfunction, impairing the differentiation and size of adipocytes and, consequently, stimulating the production of pro-inflammatory adipokines^(11)^. We highlighted that few studies with combined environmental characteristics used the structural equation modeling approach and verified their direct and indirect associations with individual characteristics, such as obesity^(12,13)^ and cardiometabolic risk^(14)^.

Environmental characteristics were greatly influenced by the rapid urbanization that occurred in middle-income countries, such as Brazil^(15)^. Thus, knowing the role of these combined characteristics can help in the development of strategies to prevent and treat adiposity-related comorbidities. Given this, we aimed to evaluate the direct and indirect associations of obesogenic and leptogenic neighborhood environments with body fat, and pro- and anti-inflammatory adipokines in Brazilian children. Our hypothesis is that obesogenic and leptogenic environments are, directly and indirectly, associated with body fat and adipokine concentrations (leptin and adiponectin).

## Methods

### Study design, setting, and sample

This cross-sectional study was conducted in 2015 among children aged 8- and 9-years residents in the urban area of Viçosa, a medium-sized city in Minas Gerais, Brazil. The city has an area of 299 km^2^, 72 244 inhabitants, of which 93·2 % live in urban areas, and the Gross Domestic Product (GDP) *per capita* is US$ 4682·22 (R$ 19 869·94)^(16)^. Viçosa is in the middle of a mountainous valley with predominantly rugged terrain. It presents a university city profile and has a more urban than agricultural economy, highlighting the role of the tertiary sector (commerce and services) as the main economic activity^(17)^.

This study came from two surveys:

1. Schoolchildren Health Assessment Survey (*Pesquisa de Avaliação da Saúde do Escolar*, PASE, in Portuguese), an investigation conducted in 2015 that aimed to assess the cardiovascular health of children aged 8 and 9 years enrolled in all schools in the urban area of Viçosa. The sample size calculation, sampling process, and non-inclusion criteria were described previously^(18)^. Briefly, 378 children were selected at school by stratified random sampling. We did not include children with health problems that altered their nutritional status or body composition; chronic use of medicine that affected the glucose and/or lipid metabolism; and when the parents/guardians could not be contacted after three attempts. For this study, we only analyzed data from children who lived in the urban area of Viçosa (*n* 367).

2. Data collection of the built environment of the urban area of Viçosa (MG), which has information on food environment, built environment, PA facilities, and neighborhood socioeconomic profile of the urban area.

Both surveys were carried out following the guidelines established in the Declaration of Helsinki and approved by the Human Research Ethics Committee of the *Universidade Federal de Viçosa* (UFV) (reference numbers 663·171/2014 and 1·821·618/2016). The Municipal Secretary of Education, the Regional Superintendent of Education and school principals approved the research. In addition, all parents/guardians signed an informed consent form.

### Body fat

The body fat percentage (%) was determined by a specialized technician using the DXA method (Lunar Prodigy Advance, GE Medical Systems Lunar, Milwaukee, WI, USA) in the diagnostic imaging sector of the UFV Health Center. At the time of exam, the children were wearing light clothing and without metal, lying in supine position on a stretcher until the equipment completed the scanning.

### Adipokines

The blood samples were collected in a 12-h fast in the clinical analysis sector of the UFV Health Center by venipuncture in the antecubital region. The serum and plasma were separated and stored in 1·5 ml Eppendorf tubes at -80ºC to measure leptin and adiponectin concentrations.

Leptin serum was analyzed by an enzyme immunoassay method (CV intra-assay <13·3 % and inter-assay <12·7 %) (KAP2281, DIAsource®, Louvain-la-Neuve, Belgium; standardized protocols from *Diagnósticos do Brasil*). Plasma adiponectin was determined by commercials ELISA sandwich kits, (coefficients of variation intra-assay <10 % and inter-assay <12 %) (human adiponectin: SEA605Hu, Cloud Clone Corp.®, Houston, TX, USA; standardized protocols from *Laboratório Especializado em Análises Científicas* – LEAC).

### Obesogenic and leptogenic environmental characteristics

The assessment of environmental characteristics has been previously described^(10)^. Briefly, an objective assessment of the food stores and the public PA facilities in the city was performed. Trained researchers, with the aid of urban census tracts maps, walked every street and, when identifying a food store and a PA facility (squares, places for walking, among others), a specific questionnaire was filled out. Four types of questionnaires, adapted from a tool developed in Brazil, were used for the objective assessment of food stores for: (a) household consumption; (b) immediate consumption; (c) mobile food vendors; and (d) farmer’s markets^(19)^. Food stores were classified according to the Technical Study ‘Mapping Food Deserts in Brazil’ by *Câmara Interministerial de Segurança Alimentar e Nutricional* (CAISAN), which considers the degree of food processing sold by store according to the NOVA classification system^(20)^. Stores whose UPF represents more than 50 % of the total products available for sale were classified as predominantly UPF stores^(21)^. To assess the public PA facilities, we used the adapted Physical Activity Resource Assessment (PARA) instrument^(22)^.

Each food store, PA facility, and household’s geographic coordinates (latitude and longitude) were obtained from addresses using the Google Maps online search service (https://www.google.com/maps?hl=en). Data on traffic accidents and crime was provided by the *Secretaria de Segurança Pública do Estado de Minas Gerais*, and its geographical coordinates (latitude and longitude). All characteristics presented in geographical coordinates were reprojected from the WGS 84 Geographic Coordinate System to the Projected Coordinate System, Universal Transverse Mercator System (UTM), 23S spindle, SIRGAS 2000 datum, using ArcGIS Pro (ESRI, Redlands, CA, USA).

The green spaces and neighborhood income were obtained from the georeferenced database at the *Instituto Brasileiro de Geografia e Estatística* ^(23)^. The presence of green spaces was identified if, on the face of the block at observation or its facing face or the median strip, there was a tree along the sidewalk and/or on a median strip that separates opposing lanes of traffic on divided roadways, even if only in part. The presence of green space was also considered when existing in areas without paving and/or without sidewalks^(23)^. The percentage of green spaces in the census tract was determined by dividing the total number of households that had the characteristic by the total number of households in the tract and multiplying by 100^(23)^. The neighborhood income was obtained by the mean household income of the census tracts from the 2010 Census (https://censo2010.ibge.gov.br/) inserted within buffer around household.

The walkability was evaluated by an index that considers residential density, presence of sidewalk (%), commercial density, presence of public lighting (%), and the density of intersections between streets (connectivity), as previously described^(24)^.

To assess the obesogenic and leptogenic environmental characteristics around households, we used 400 m (0·25 miles) road network buffer as the neighborhood unit, corresponding to a 5 min’ walk^(25,26)^. Within each buffer, the absolute density was calculated. Then, the density per 1000 inhabitants was obtained for predominantly UPF food stores, PA facilities, traffic accidents, and crime. For green spaces, neighborhood income, walkability, the census tracts averages inserted within a buffer around households were obtained. Then, all environmental characteristics were categorized according to the 50th percentiles.

### Mediating and adjusting variables

Through a semi-structured questionnaire, individual and family data, such as sex, per capita household income (US$), maternal education (years), receipt of government benefit (yes/no), and PA before and after school (yes/no), were collected. *Per capita* income was calculated by dividing the total household income by the number of dependent residents on that household income.

The mother’s weight and height were measured by an electronic digital scale with a capacity of 150 kg and a sensitivity of 50 g (Tanita^®^, model BC 553, Arlington Heights, IL, USA) and a vertical stadiometer, divided into centimeters and subdivided into millimeters (Alturexata^®^, Belo Horizonte, MG, Brazil), respectively. Then, the mother’s BMI was calculated.

UPF consumption was estimated using the average of three 24-h dietary recalls (24HR) obtained on non-consecutive days, including 1 weekend day. The data were collected from the parents/guardian and the child simultaneously. All 24HR were conducted by trained dietitians using the 5-step multiple-pass method^(27)^. To improve portion size estimation and increase data reliability, during the interviews the participants had access to kitchen measuring utensils and food photographic records^(28)^. The software Diet Pro® 5i, version 5.8, was used to record food consumption information. To analyze the food composition, the *Tabela Brasileira de Composição de Alimentos* (TACO)^(29)^ was used preferentially, following by the US Department of Agriculture’s of Food Composition Database^(30)^. Measures utilizing kitchen utensils were converted into grams (g), milligrams (mg), or milliliters (ml) to estimate consumption of total energy (kcal) and UPF, defined according to the NOVA classification^(20)^. The mean percentage of UPF energy contribution from the three 24HR was calculated.

### Data analysis and structural equation modeling

Descriptive analysis was performed by calculating frequency distributions for categorical variables and median and interquartile range (IQR) for continuous variables using the statistical software Stata^®^ version 14 (StataCorp., LP). The normality of numerical variables was assessed using the Shapiro-Wilk test. The Mann-Whitney test and Pearson’s chi-squared test compared numerical and categorical variables according to sex, respectively. The level of significance for all statistical tests was set at 0·05.

**Fig. 1:**
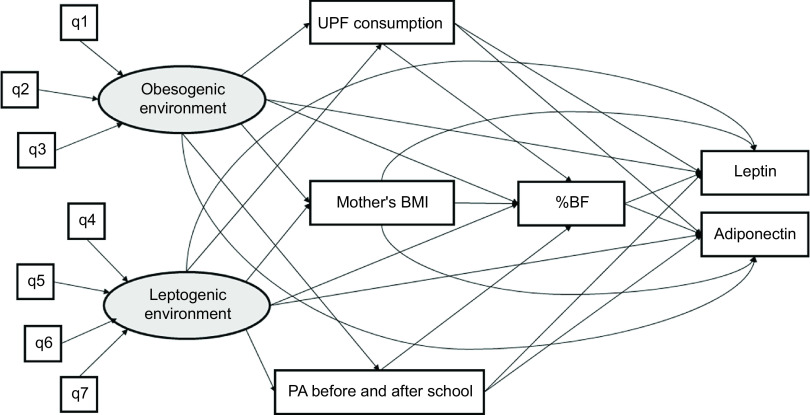
Initial theoretical model of structural equations hypothesized for the associations of obesogenic and leptogenic environments with % body fat and adipokine concentrations in children. Viçosa, Minas Gerais, Brazil, 2015. Abbreviations: %BF: %body fat; BMI: body mass index; PA: physical activity; q1: predominantly ultra-processed food stores; q2: traffic accidents; q3: crime; q4: walkability; q5: physical activity facilities; q6: green spaces; q7: neighborhood income; UPF: ultra-processed food. The observed variables were presented in rectangular shapes and the latent variables in circular shapes. Models with % body fat, adipokines, ultra-processed food consumption, and physical activity before and after school as outcome were adjusted for child’s sex and socioeconomic status. Models with mother’s BMI as outcome were adjusted for socioeconomic status

Structural equation modeling was conducted in the software Mplus version 8.2 (Muthén & Muthén). The initial theoretical hybrid model of structural equations was composed of obesogenic and leptogenic environments in a distal position, related to the UPF consumption, mother’s BMI, and PA before and after school. In the model, the obesogenic and leptogenic environments might directly associate with %BF and pro- and anti-inflammatory adipokines. Furthermore, the environments may be indirectly associated with body fat, being mediated by the UPF consumption, mother’s BMI, and PA before and after school. Therefore, body fat was also considered a mediating variable in the relationship between the obesogenic/leptogenic environments and adipokines. According to the literature review, the obesogenic environment was initially composed of the variables predominantly UPF stores, traffic accidents, and crime. In contrast, the leptogenic environment would consist of walkability, PA facilities, green spaces, and neighborhood income (Fig. 1).

Latent variables were validated, with exploratory and confirmatory analyses. The exploratory evaluation was performed through principal component analysis (PCA), obtaining the Kaiser-Meyer-Olkin (KMO) index with values between 0·5 and 1·0 considered acceptable^(31)^. For the construction of latent variables, a factor loading greater than 0·4 and a *P* < 0·05 were considered to indicate that the correlation between the observed variable and the latent variable had a moderately high magnitude^(32)^. After confirmatory analyses, latent variables for the obesogenic and leptogenic environments were obtained from observed environmental variables and categorized according to the 50th percentile of the sample:

• Obesogenic environment: predominantly UPF stores, traffic accidents, crime, and walkability.

• Leptogenic environment: public PA facilities, green spaces, and neighborhood income.

Estimates were obtained using the Weighted Least Squares Mean and Variance Adjusted (WLSMV), which is applied to categorical variables, robust in the absence of normality and imputes missing data using Full Information Maximum Likelihood (FIML) estimation^(32)^. Theta parameterization was used to control for differences in residual variances^(33)^.

Models with body fat, adipokines, UPF consumption, and PA before and after school as outcomes were adjusted for the child’s sex and socioeconomic status (latent variable composed of per capita income, maternal education, and government benefit). Models with the mother’s BMI as outcome were adjusted for socioeconomic status. The latent variable of socioeconomic status was also validated.

To assess the quality of fit of the hybrid structural equation model, the following criteria were considered: chi-squared test with *P* > 0·05; root-mean-square error of approximation (RMSEA) with values less than 0·08; values of the comparative fit index (CFI) and the Tucker Lewis index (TLI) approximately or greater than 0·95; standardized root mean square residual (SRMR) less than 1^(34)^.

The modindices command was used to calculate the modification indices, to obtain suggestions for changes in the initial hypotheses. To carry out the changes, theoretical plausibility and modification index values equal to or greater than 10 were considered^(32,34)^. All direct and indirect associations of latent and observed variables were evaluated in the final model.

Sensitivity analysis was also performed to verify the robustness of the associations in all models, with the variables being tested in different ways: 1^st^: body fat was categorized according to tertiles; 2^nd^: body fat was replaced by % android fat, being tested continuously and categorized into tertiles; 3^rd^: adipokines were categorized according to quintiles; 4^th^: *per capita* income (in tertiles) and maternal education (<4 years, 5–8 years, 9–11 years and ≥ 12 years) were also categorically tested.

## Results

### Sample characterization

We evaluated 367 children and their respective mothers, with age mean 8·5 ± 0·5 years; 51·8 % were girls, 22·9 % were government program beneficiaries. The median of body fat and leptin and adiponectin concentrations were 22·7 %, 1·7 ng/ml, and 2·4 µg/dl, respectively. According to sex, female children had higher body fat, leptin concentration, and less PA before and after school (Table 1).

**Table 1:** Body fat, adipokine concentrations, socioeconomic, and health behaviors in children, according to sex. Viçosa, Minas Gerais, Brazil, 2015

Characteristics	Overall (*n* 367)		Female (*n* 190)		Male (*n* 177)		*P*-value
Body fat (%)	22·7	16·1, 32·1	25·3	19·1, 34·7	19·5	13·4, 29,9	**<0·001** ^*^
Leptin (ng/ml)	1·7	0·4, 6·4	2·5	0·6, 10·1	1·4	0·3, 4·5	**0·002** ^*^
Adiponectin (µg/ml)	11·4	8·1, 17·0	11·6	7·8, 17·3	11·3	8·5, 16·3	0·86
*Per capita* income (R$)	500	315, 834	500	332, 900	500	300, 800	0·66
Maternal education (years)	11	8, 11	11	8, 11	11	7, 11	0·78
Government program beneficiary							
Yes	84	22·9 %	42	22·1 %	42	23·7 %	0·71
No	283	77·1 %	148	77·9 %	135	76·3 %	
Mother’s BMI (kg/m^2^)	25·8	22·8, 29·7	25·7	22·9, 29·3	25·8	22·7, 30·3	0·71
UPF consumption (% energy)	45·2	34·3, 54·5	45·0	33·7, 53·5	46·4	37·6, 55·7	0·20
PA before and after school							
Yes	126	34·4 %	54	28·6 %	72	40·7 %	**0·02** ^†^
No	240	65·6 %	135	71·4 %	105	59·3 %	

Abbreviations: BMI: body mass index; PA: physical activity; UPF: ultra-processed food.

* Mann-Whitney test.

† Pearson’s chi-squared test.

Bold values (*P* < 0·05).

Data presented as median (interquartile range) for numerical variables or *n* (%) for categorical variables.

### Validation of latent variables

**Fig. 2:**
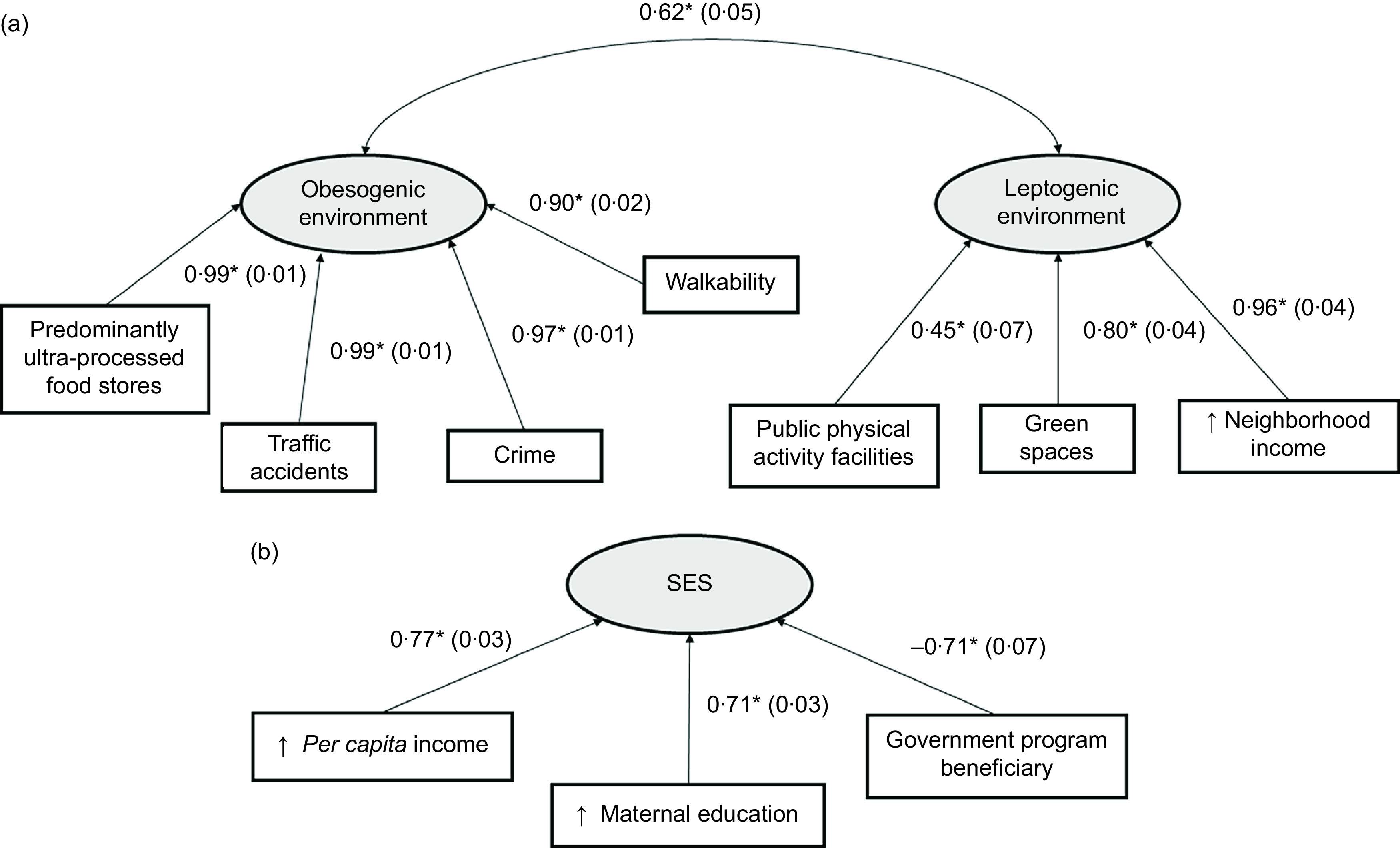
Validation of latent variables (a) obesogenic and leptogenic environments, and (b) socioeconomic status. Abbreviations: SES: socioeconomic status. Data presented as factor loading (SE). The observed variables were presented in rectangular shapes and the latent variables in circular shapes. For environmental variables, Kaiser-Meyer-Olkin (KMO) index = 0·80, % variance of 1^st^ component (obesogenic environment): 47·7 %, % variance of 2^nd^ component (leptogenic environment): 25·5 %, % variance total = 73·2 %. For socioeconomic status, Kaiser-Meyer-Olkin (KMO) index = 0·59, and % variance total = 58·1 %. *P < 0·001

We performed an exploratory analysis of the available environmental variables and identified two principal components, presenting a satisfactory KMO (0·80). The first component contributed to 47·7 % of the variance and the second contributed to 25·5 %, totaling 73·2 % of the variance. The constructs were satisfactory in convergent and discriminatory validities, in which each construct had different measurement, and the correlation between them was significant, but less than 0·90 (0·62, *P* < 0·001). In general, the first component was composed of variables that characterize an obesogenic environment, while the second component was composed of variables that characterize a leptogenic environment (Fig. 2(a)).

From the socioeconomic variables, we obtained only one principal component, which presented a satisfactory KMO (0·59) and convergent validity, and explained 58·1 % of the total variance (Fig. 2(b)).

### Structural equation models

**Fig. 3:**
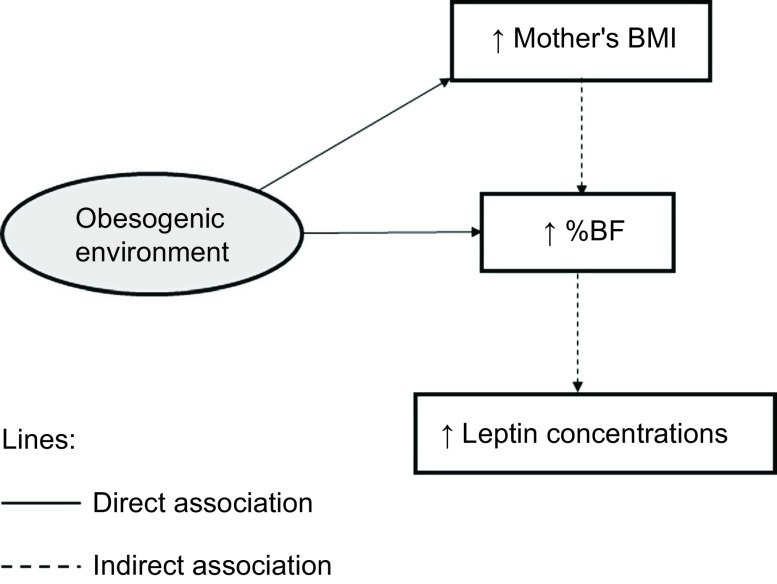
Direct and indirect associations of the obesogenic neighborhood environment with body fat and adipokine concentrations in Brazilian children. Abbreviations: % BF: % body fat; BMI: body mass index. Solid lines represent direct associations and dotted lines represent indirect associations

The obesogenic environment was directly associated with the mother’s BMI (*β*: 0·24, *P* = 0·02) and the child’s body fat (*β*: 0·19, *P* = 0·02). We identified an indirect association of the obesogenic environment with the child’s body fat (*β*: 0·08, *P* = 0·04), being mediated by the mother’s BMI (*β*: 0·09, *P* = 0·02). We also observed an indirect association of the obesogenic environment with leptin concentrations (*β*: 0·17, *P* = 0·001), being mediated by child’s body fat (*β*: 0·10, *P* = 0·03), as well by mother’s BMI and child’s body fat (*β*: 0·05, *P* = 0·02) (Table 2; Fig. 3).

**Table 2:** Standardized coefficients (*β*) of the direct and indirect associations of obesogenic neighborhood environment with body fat and adipokine concentrations in children. Viçosa, Minas Gerais, Brazil, 2015

Pathways	*β*	se	*P*-value
**Direct associations**			
Obesogenic environment → UPF consumption	0·08	0·09	0·36
Obesogenic environment → mother’s BMI	0·24	0·10	**0·02**
Obesogenic environment → PA before and after school	−0·05	0·11	0·65
Obesogenic environment → % BF	0·19	0·08	**0·02**
Obesogenic environment → leptin	−0·01	0·06	0·85
Obesogenic environment → adiponectin	0·00	0·09	0·99
**Indirect associations**			
Obesogenic environment → % BF	0·08	0·04	**0·04**
Obesogenic environment → UPF consumption → % BF	−0·01	0·01	0·50
Obesogenic environment → mother’s BMI → % BF	0·09	0·04	**0·02**
Obesogenic environment → PA before and after school → % BF	0·00	0·01	0·77
Obesogenic environment → leptin	0·17	0·05	**0·001**
Obesogenic environment → UPF consumption → leptin	0·00	0·01	0·50
Obesogenic environment → mother’s BMI → leptin	0·01	0·02	0·53
Obesogenic environment → PA before and after school → leptin	0·00	0·00	0·88
Obesogenic environment → % BF → leptin	0·10	0·05	**0·03**
Obesogenic environment → UPF consumption → % BF → leptin	0·00	0·00	0·50
Obesogenic environment → mother’s BMI → % BF → leptin	0·05	0·02	**0·02**
Obesogenic environment → PA before and after school → % BF → leptin	0·00	0·00	0·77
Obesogenic environment → adiponectin	−0·04	0·02	0·05
Obesogenic environment → UPF consumption → adiponectin	0·00	0·01	0·68
Obesogenic environment → mother’s BMI → adiponectin	−0·01	0·02	0·64
Obesogenic environment → PA before and after school → adiponectin	0·00	0·01	0·94
Obesogenic environment → % BF → adiponectin	−0·02	0·02	0·16
Obesogenic environment → UPF consumption → % BF → adiponectin	0·00	0·00	0·53
Obesogenic environment → mother’s BMI → % BF → adiponectin	−0·01	0·01	0·15
Obesogenic environment → PA before and after school → % BF → adiponectin	0·00	0·00	0·78
**Total associations**			
Obesogenic environment → % BF	0·27	0·09	**0·002**
Obesogenic environment → leptin	0·16	0·07	**0·03**
Obesogenic environment → adiponectin	−0·04	0·09	0·64

Abbreviations: % BF: % body fat; BMI: body mass index; PA: physical activity; se: standard error; UPF: ultra-processed food; *β*: standard coefficient.

Structural equation modeling. Model fit information: *X* ^2^ = 206·88, *P* < 0·001; Root Mean Square of Approximation: 0.06, 90 % CI (0 05, 0·07); Comparative Fit Index = 0·99; Tucker Lewis Index = 0·99; Standardized Root Mean Square Residual: 0·06.

Models with % body fat, adipokines, ultra-processed food consumption, and physical activity before and after school as outcomes were adjusted for child’s sex and socioeconomic status. Models with mother’s BMI as outcome were adjusted for socioeconomic status.

Bold values (*P* < 0·05).

The direct and indirect associations of the leptogenic environment with body fat and adipokine concentrations were not observed (Table 3).

**Table 3:** Standardized coefficients (*β*) of the direct and indirect associations of leptogenic neighborhood environment with body fat and adipokine concentrations in children. Viçosa, Minas Gerais, Brazil, 2015

Pathways	*β*	se	*P*-value
**Direct associations**			
Leptogenic environment → UPF consumption	0·10	0·13	0·45
Leptogenic environment → mother’s BMI	−0·07	0·15	0·62
Leptogenic environment → PA before and after school	0·08	0·15	0·58
Leptogenic environment → % BF	−0·21	0·12	0·09
Leptogenic environment → leptin	−0·12	0·11	0·27
Leptogenic environment → adiponectin	−0·18	0·13	0·17
**Indirect associations**			
Leptogenic environment → % BF	−0·03	0·05	0·57
Leptogenic environment → UPF consumption → % BF	−0·01	0·01	0·45
Leptogenic environment → mother’s BMI → % BF	−0·03	0·05	0·61
Leptogenic environment → PA before and after school → % BF	0·00	0·01	0·75
Leptogenic environment → leptin	−0·14	0·07	0·07
Leptogenic environment → UPF consumption → leptin	0·00	0·01	0·58
Leptogenic environment → mother’s BMI → leptin	0·00	0·01	0·66
Leptogenic environment → PA before and after school → leptin	0·00	0·01	0·88
Leptogenic environment → % BF → leptin	−0·12	0·07	0·08
Leptogenic environment → UPF consumption → % BF → leptin	0·00	0·01	0·46
Leptogenic environment → mother’s BMI → % BF → leptin	−0·02	0·03	0·61
Leptogenic environment → PA before and after school → % BF → leptin	0·00	0·01	0·75
Leptogenic environment → adiponectin	0·03	0·03	0·25
Leptogenic environment → UPF consumption → adiponectin	0·00	0·01	0·65
Leptogenic environment → mother’s BMI → adiponectin	0·00	0·01	0·74
Leptogenic environment → PA before and after school → adiponectin	0·00	0·01	0·94
Leptogenic environment → % BF → adiponectin	0·03	0·02	0·23
Leptogenic environment → UPF consumption → % BF → adiponectin	0·00	0·00	0·49
Leptogenic environment → mother’s BMI → % BF → adiponectin	0·00	0·01	0·62
Leptogenic environment → PA before and after school → % BF → adiponectin	0·00	0·00	0·76
**Total associations**			
Leptogenic environment → % BF	−0·23	0·13	0·07
Leptogenic environment → leptin	−0·26	0·13	0·05
Leptogenic environment → adiponectin	−0·15	0·13	0·23

Abbreviations: % BF: % body fat; BMI: body mass index; PA: physical activity; se: standard error; UPF: ultra-processed food; *β*: standard coefficient.

Structural equation modeling. Model fit information: *X* ^2^ = 206·88, *P* < 0·001; Root Mean Square of Approximation: 0·06, 90 % CI (0 05, 0·07); Comparative Fit Index = 0·99; Tucker Lewis Index = 0·99; Standardized Root Mean Square Residual: 0·06.

Models with % body fat, adipokines, ultra-processed food consumption, and physical activity before and after school as outcomes were adjusted for child’s sex and socioeconomic status. Models with mother’s BMI as outcome were adjusted for socioeconomic status.

In general, the models showed good quality of fit, satisfying most of the criteria: chi-squared test: 206·88, *P* < 0·001; RMSEA: 0·06, 90 % CI: 0·05, 0·07, *P* = 0·05; CFI: 0·99; TLI: 0·99; SRMR: 0·06.

When the sensitivity analysis was performed, considering different categorization forms of variables and the replacement of body fat by android fat, the same results were observed (data not shown).

## Discussion

Our findings supported the hypothesis that the obesogenic neighborhood environment has a direct and indirect association with children’s body fat, with the mother’s BMI as the main mediator. Furthermore, the obesogenic environment was indirectly associated with leptin concentrations, with the mother’s BMI and child´s body fat as mediators.

The relationship between the obesogenic environment and child health has been investigated, however the studies were conducted in high-income countries and evaluated the isolated environmental characteristics^(35,36)^. In this study, we have two innovations. First, it is known that middle-income countries, such as Brazil, have a different development and urbanization profile compared to high-income countries^(37)^. Second, when we used the latent variables of obesogenic and leptogenic environments, we considered jointly the association of all variables that composed them with outcomes.

The obesogenic environment had a direct association with the child’s body fat. Although some evidence did not confirm this relationship^(12,38)^, other studies showed similar results^(13,39)^. Among Portuguese schoolchildren, there was a direct association of an unsafety environment (excessive traffic and intersections, high crime rates) with the increase in the BMI *Z* score^(39)^. In addition, the neighborhood socioeconomic status (lower education, poverty level, unemployment rates, lower per capita income, and vacant housing) and social disorganization (single-parent households and the proportion of those divorce or separated for both males and females) had a direct association with obesity among American adolescents^(13)^. In adolescents from the United States, the access to PA facilities (recreation center, sidewalks/walking paths, and parks/playgrounds) was directly associated for reducing obesity^(39)^.

Additionally, our results showed that the mother’s BMI was the main mediator in the association of the obesogenic environment with body fat and leptin concentrations. A study conducted among children from Seattle and San Diego, United States, showed a direct association between the obesogenic environment, obesity, and parental obesity^(40)^. In Brazilian children, the mother’s BMI can be a proxy for the child’s body adiposity due to genetic and unhealthy behavioral factors, considering eating habits and PA^(41,42)^, and it was also a mediator in the relationship between the obesogenic environment and a higher cardiometabolic risk^(14)^. These findings suggest that strategies to reduce the obesogenic environment around households may impact the child’s body fat as well as their parents, who are mainly responsible for the acquisition of food and encourage the practice of physical and leisure activities by children. However, we did not find studies investigating these relationships with leptin concentrations in childhood.

The obesogenic and leptogenic environments were not directly associated with UPF consumption and PA before and after school. Furthermore, these behavioral characteristics were not mediators of the association with body fat and adipokines. Similarly, among Portuguese, there was a lack of mediation of characteristics related to PA between favorable/unsafety environments and a child’s BMI^(39)^. It is important to consider that other factors can be related with these behavioral characteristics beyond the community environment, such as shopping preferences, the modes of transportation, and home environment^(43)^.

This is the first investigation that evaluated the association of the obesogenic and leptogenic environments with adiposity-related inflammation markers. There is no evidence of mediating role of mother’s BMI on a child’s inflammatory markers; however, it is known that obesity is the main mediator between food consumption and low-grade inflammation^(44)^. From the knowledge about the association between adipokines and cardiometabolic outcomes, such as insulin resistance markers and blood pressure^(45)^, it is necessary to assess the relationship between the environment and markers beyond the traditional body adiposity measures. Evidence has shown associations of the obesogenic environment with the child’s BMI^(43)^, an easy-to-use and low-cost measure. Nevertheless, phenotyping of obesity has been proposed considering other measures/markers, such as those obtained by reference methods (DXA) and pro- and anti-inflammatory adipokines^(46)^.

We emphasize that walkability was part of the latent variable obesogenic environment, with a direct factor loading, even initially being part of the leptogenic environment in the theoretical model. Walkability quantifies the safety and desirability of the walking routes^(47)^, being a measure of urban design in connecting places and facilitating PA^(48)^. However, in urban areas, greater walkability was associated with a higher BMI among children^(49)^. We used a walkability index proposed to the city studied that considers important characteristics that favor walking (residential and commercial densities, intersections between roads, the presence of sidewalks and public lighting)^(26)^. The city is more walkable in the downtown; however, this region also has a higher density of UPF stores, traffic accidents, and crime. In this sense, further studies are needed to understand the role of walkability on health outcomes, considering different aspects of urbanization.

The leptogenic environment was not associated with adiposity and related inflammation in children. This lack of association may be attributed to the urban environment of Viçosa, since leptogenic characteristics coexist with obesogenic ones in the downtown area^(10)^. Additionally, these outcomes are derived from a multi-complex model, involving not only the environment, but also family and individual-child factors^(8)^. In this sense, further studies are needed to investigate the environmental characteristics that promote healthy habits in childhood. This evidence may help health planners to develop strategies, jointly with other sectors of urban planning, to improve children’s quality of life and, consequently, preventing and/or reducing obesity and adiposity-related inflammation.

This work has several strengths. First, this study is a pioneer in evaluating the associations of obesogenic and leptogenic environments with children’s health in a middle-income country. Second, the structural equation modeling approach allowed us to assess the direct and indirect associations between the environment and unhealthy outcomes. Third, we conducted a sensitivity analysis, testing android fat and other forms of variable categorization, which confirmed these results. Also, this is the first study to evaluate the associations of obesogenic and leptogenic environments with pro- and anti-inflammatory adipokines and the use body fat obtained from a reference method (DXA). Finally, we highlight that, although some environmental characteristics were obtained from the 2010 Census database, the information has high quality and allows for the replication and comparison of results between different Brazilian cities.

As limitation, this investigation was carried out in a Brazilian medium-sized city, which is largely made up of a young population (university students), and justifies a greater concentration of activities and services in the downtown area and near to the university^(10)^. Therefore, extrapolation of these results should be cautious, and studies in different geographic locations are necessary to better understand the relationship between environmental characteristics and adiposity-related inflammation in children. Furthermore, the cross-sectional design does not allow the establishment of a cause-effect relationship. As the urban environment is dynamic, longitudinal studies must be conducted to verify the long-term effect of environmental characteristics on child health.

We conclude that the obesogenic neighborhood environment was directly associated with body fat and the mother’s BMI, and indirectly with leptin concentrations (pro-inflammatory marker) in Brazilian children, mediated by the child’s body fat and mother’s BMI. Therefore, encouraging the development of urban environments that promote healthier food and lifestyle choices can be one of the steps to prevent and/or reduce these childhood comorbidities.
